# The role of the secondary cell wall in plant resistance to pathogens

**DOI:** 10.3389/fpls.2014.00358

**Published:** 2014-08-05

**Authors:** Eva Miedes, Ruben Vanholme, Wout Boerjan, Antonio Molina

**Affiliations:** ^1^Centro de Biotecnología y Genómica de Plantas, Universidad Politécnica MadridMadrid, Spain; ^2^Departamento Biotecnología, Escuela Técnica Superior Ingenieros Agrónomos, Universidad Politécnica MadridMadrid, Spain; ^3^Department of Plant Systems Biology, VIB (Flanders Institute for Biotechnology)Gent, Belgium; ^4^Department of Plant Biotechnology and Bioinformatics, Ghent UniversityGent, Belgium

**Keywords:** cell wall, plant pathogen, plant immunity, xylan, cellulose, lignin

## Abstract

Plant resistance to pathogens relies on a complex network of constitutive and inducible defensive barriers. The plant cell wall is one of the barriers that pathogens need to overcome to successfully colonize plant tissues. The traditional view of the plant cell wall as a passive barrier has evolved to a concept that considers the wall as a dynamic structure that regulates both constitutive and inducible defense mechanisms, and as a source of signaling molecules that trigger immune responses. The secondary cell walls of plants also represent a carbon-neutral feedstock (lignocellulosic biomass) for the production of biofuels and biomaterials. Therefore, engineering plants with improved secondary cell wall characteristics is an interesting strategy to ease the processing of lignocellulosic biomass in the biorefinery. However, modification of the integrity of the cell wall by impairment of proteins required for its biosynthesis or remodeling may impact the plants resistance to pathogens. This review summarizes our understanding of the role of the plant cell wall in pathogen resistance with a focus on the contribution of lignin to this biological process.

## INTRODUCTION

In their natural environments, plants are under continuous threat of biotic stresses caused by pathogenic bacteria, fungi, viruses, and oomycetes, that compromise plant survival and reproduction (Panstruga et al., 2009). Given that green plants are the ultimate source of energy for most other organisms, it is not surprising that plants have evolved a plethora of resistance mechanisms which are either constitutively present or induced after pathogen attack (Glazebrook, 2005; Panstruga et al., 2009). An important defense element common to all plants is the cell wall.

All the plant cells that are in developmental expansion have a constantly remodeled primary cell wall that mainly consists of carbohydrate-based polymers (classified as cellulose, hemicelluloses and pectins) and hydroxyproline-rich *O*-glycoproteins, such as extensins and arabinogalactan proteins (AGPs; Carpita and McCann, 2000). In addition, those cells that have completed their cellular expansion and need to reinforce their structure for functional reasons (e.g., to form vessel or fiber cells) generate a secondary cell wall that is mainly composed of cellulose, hemicelluloses (mostly xylans) and lignin (Cosgrove, 2005; Sarkar et al., 2009). Besides having multiple essential functions during plant development, plant cell walls also play important roles in preventing pathogen invasion. First, cell walls act as a passive barrier; local or extensive breakdown of the wall matrix is typically required for the progression of pathogen infection (Cantu et al., 2008; Hematy et al., 2009). Second, the cell wall is a reservoir of antimicrobial compounds, which are released during cell wall degradation (García-Olmedo et al., 2001; Schulze-Lefert, 2004; Vorwerk et al., 2004). Moreover, plants have a dedicated cell wall integrity (CWI) maintenance mechanism similar to that existing in fungi, which initiates responses to regulate CWI during plant development and in response to external stimuli (Wolf et al., 2012; Engelsdorf and Hamann, 2014). Impairment of CWI by pathogen attack or wounding results in the release of plant signaling molecules, the so-called Damage-Associated Molecular Patterns (DAMPs; Vorwerk et al., 2004; Cantu et al., 2008). DAMPs can modulate plant innate immune responses upon recognition by plant Pattern Recognition Receptors (PRRs), through molecular mechanisms that are similar to those regulating the activation of immune responses by Pathogen-Associated Molecular Patterns (PAMPs) derived from microbial pathogens (reviewed by Dodds and Rathjen, 2010; Macho and Zipfel, 2014; Malinovsky et al., 2014). The recognition of DAMPs and PAMPs by PRRs activates protein kinase cascades, which regulate downstream immune responses that can lead, among others, to cell-wall reinforcement (Ringli, 2010; Ferrari et al., 2013; Engelsdorf and Hamann, 2014; Malinovsky et al., 2014). Notably, in addition to its role in protecting plants against infection, the plant cell wall can also act as a source of nutrients from the pathogen point of view, thereby promoting pathogen growth and development (Cantu et al., 2008; Hematy et al., 2009).

Cell walls are also considered as a valuable feedstock for the production of second generation biofuels and bio-based chemicals. These so-called lignocellulosic feedstocks can either derive from agricultural and industrial practices, such as maize stover, straw and sugarcane bagasse, or from dedicated crops, such as fast growing grasses and trees, grown for the purpose of generating large volumes of lignocellulosic biomass. In both cases, engineering cell wall composition is a promising strategy to ease lignocellulosic biomass conversion toward fuels and chemicals in industrial processes (Simmons et al., 2010; Ong et al., 2014). Importantly, the changes in cell-wall composition needed for industrial biomass processing should not conflict with the principal biological roles of the cell wall as a supportive and protective structure. Indeed, some cell wall modifications result in negative repercussions on biomass yield (Bonawitz and Chapple, 2013). A better understanding of the processes underlying the yield-penalty in plants with modified cell walls has led to successful engineering strategies to recover the biomass yield, while maintaining the anticipated cell wall modifications (Petersen et al., 2012; Yang et al., 2013; Bonawitz et al., 2014). Likewise, cell wall modifications should not have negative repercussions on crop susceptibility toward pathogens. Clearly, a better understanding of the processes underlying the interactions between pathogens and the cell wall will support the development of plants with optimized lignocellulosic characteristics, without negatively affecting disease resistance.

A relatively large number of studies have described the influence of plant cell-wall modifications on pathogen infection (Cantu et al., 2008; Bellincampi et al., 2014; Malinovsky et al., 2014). Contra-intuitively, “weakening” the cell wall by knocking out essential genes involved in cell-wall biosynthesis sometimes leads to enhanced resistance toward specific pathogens. In this review, we summarize the consequences of secondary cell-wall modifications on pathogenic infection and link them with our current knowledge on the role of the cell wall in plant resistance to pathogens. Because lignin is both stress-induced and developmentally deposited in the secondary thickened cell wall and because it is a major target for lignocellulosic biomass engineering, we have put special emphasis on the effect of altering lignin amount and composition on pathogen infection and spread.

## THE EFFECT OF ALTERING CELL WALL POLYSACCHARIDES ON RESISTANCE TO PATHOGENS

The contribution of the secondary cell wall to plant immunity has been mainly demonstrated through the characterization of plant mutants impaired in secondary wall composition (Cantu et al., 2008; Underwood, 2012). For instance, the resistance of a set of *Arabidopsis thaliana* (*Arabidopsis* herein) mutants defective in cellulose synthase (CESA) subunits required for secondary cell wall formation (i.e., CESA4, CESA7, and CESA8) toward a series of pathogens has been tested. These mutants make less cellulose, which results in collapsed xylem vessels, and therefore they are called *ir*regular *x*ylem mutants (*irx5*, *irx3*, and *irx1*, respectively). These mutants showed enhanced resistance to different pathogens, including the necrotrophic fungi *Plectosphaerella cucumerina* and *Botrytis cinerea*, the vascular bacterium *Ralstonia solanacearum*, and the biotrophic bacterium *Pseudomonas syringae* (**Table 1**; Hernández-Blanco et al., 2007). In line with these results, an *Arabidopsis* mutant defective in the MYB46 transcription factor that directly regulates the expression of genes required for secondary cell wall formation, including lignin and cellulose biosynthesis (among which *CESA4, CESA7*, and *CESA8*), also showed enhanced resistance to necrotrophic fungi (Ramírez et al., 2011). The disease resistance phenotype of *irx1*, *irx3*, *irx5*, and *myb46* mutants was in part explained by the constitutive activation of plant immune responses rather than by alterations of the passive wall barrier. In these mutants, the abscisic acid signaling pathway was constitutively active and antimicrobial peptides and tryptophan-derived metabolites accumulated to a higher extent than in wild-type plants (Hernández-Blanco et al., 2007; Sánchez-Vallet et al., 2010). Plant resistance to pathogens is also altered in *Arabidopsis* mutants affected in CESAs subunits required for cellulose biosynthesis of the primary cell wall, such as the CESA3 defective *isoxaben resistant* (*ixr1*)/*constitutive expression of VSP* (*cev1*) mutants (Ellis et al., 2002). The *ixr1/cev1* mutant alleles are more resistant than wild-type plants to *B. cinerea*, *P. syringae*, and *Erysiphe cichoracearum* (Ellis et al., 2002), whereas their resistance to *R. solanacearum* and *P. cucumerina* does not differ from that of wild-type plants, which contrasts with the resistance phenotype of the secondary cell wall cellulose mutants, *irx1, irx3* and *irx5* (Hernández-Blanco et al., 2007). In *ixr1/cev1* plants, the ethylene and jasmonic acid, but not the abscisic acid signaling pathway, are constitutively activated. These results with *Arabidopsis cesA* mutants illustrate that specific immune responses can be activated by alteration of the CWI of either the primary or the secondary wall (Ellis et al., 2002; Hernández-Blanco et al., 2007).

**Table 1:** Resistance phenotype of plants with alterations in secondary cell wall structure/composition.

Gene name (mutant/transgenic)	Plant species	Pathogen tested	Phenotype^1^	Reference
*CESA4, CESA7, CESA8 (irx5, irx3, irx1)*	*Arabidopsis thaliana*	*Plectosphaerella cucumerina*, *Ralstonia solanacearum*, *Botrytis cinerea, Pseudomonas syringae*	R	Hernández-Blanco et al. (2007)
*WAT (wat1)*	*Arabidopsis thaliana*	*R. solanacearum, P. cucumerina*, *Xanthomonas campestris*, *Verticillium dahlia, V. alboatrum*	R	Denancé et al. (2013)
*DET3 (det3)*	*Arabidopsis thaliana*	*P. cucumerina*	R	Delgado-Cerezo et al. (2012)
*XYL1 (xyl1-2)*	*Arabidopsis thaliana*	*P. cucumerina*	R	Delgado-Cerezo et al. (2012)
*RWA2 (rwa2)*	*Arabidopsis thaliana*	*B. cinerea*	R	Manabe et al. (2011)
*IRX6 (irx6)*	*Arabidopsis thaliana*	*P. cucumerina*	R	Delgado-Cerezo et al. (2012)
*MYB46 (myb46)*	*Arabidopsis thaliana*	*B. cinerea*	R	Ramírez et al. (2011)
*PAL (pal1/2/3/4)*	*Arabidopsis thaliana*	*P. syringae*	S	Huang et al. (2010)
*COMT (comt1)*	*Arabidopsis thaliana*	*X. campestris*, *P. syringae*, *B. cinerea*, *Blumeria graminis*, *Alternaria brassicicola*	S	Quentin et al. (2009)
		*Hyaloperonospora arabidopsidis*	R^2^	Quentin et al. (2009)
*F5H1 (f5h1)*	*Arabidopsis thaliana*	*Sclerotinia sclerotiorum*	S	Huang et al. (2009)
*F5H1 (fah1-2)*	*Arabidopsis thaliana*	*Verticillium longisporum*	S	König et al. (2014)
*AXE (35S::AnAXE)^3^*	*Arabidopsis thaliana*	*B. cinerea*	R	Pogorelko et al. (2013)
*PAL (35S::PvPAL2)^3^*	*Nicotiana tabacum*	*Cercospora nicotianae*	R	Shadle et al. (2003)
*PAL (35S::ShPAL)^3^*	*Nicotiana tabacum*	*Phytophthora parasitica*, *C. nicotianae*	R	Way et al. (2002, 2011)
*COMT (comt)*	*Nicotiana tabacum*	*Agrobacterium tumefaciens*	R	Maury et al. (2010)
*HCT (HCT antisense)*	*Medicago sativa*	*Colletotrichum trifolii*	R	Gallego-Giraldo et al. (2011b)
*PAL, CCoAOMT, COMT, CAD (RNAi)^4^*	*Triticum monococcum*	*Blumeria graminis f. sp. tritici*	S	Bhuiyan et al. (2009)
*CAD (RNAi)^4^*	*Linum usitatissimum*	*Fusarium oxysporum*	S	Wróbel-Kwiatkowska et al. (2007)
*CAD (bmr6)*	*Sorghum bicolor*	*Fusarium thapsinum*, *F. proliferatum*, *F. verticillioides*, *Alternaria alternata*	R	Funnell-Harris et al. (2010)
*COMT (bmr12)*	*Sorghum bicolor*	*F. thapsinum*, *F. proliferatum*, *F. verticillioides*, *A. alternata*	R	Funnell-Harris et al. (2010)
*AXE (35S::AnAXE)^3^*	*Brachypodium distachyon*	*Bipolaris sorokiniana*	R	Pogorelko et al. (2013)

1 R; enhanced resistance compared with wild-type plants; S, enhanced susceptibility compared with wild-type plants.

2 Enhanced resistance to downy mildew was not correlated with increased plant defense responses in comt1 mutant, but coincided with a higher frequency of oomycete sexual reproduction within mutant tissues.

3 Genes from Aspergillus nidulans (An), Phaseolus vulgaris (Pv) and Stylosanthes humilis (Sh).

4 RNA interference constructs were made by a combined ligation/recombination (LR) method using plasmid pIPKTA30N as the final GATEWAY destination vector (Bhuiyan et al., 2009) and a self-complementary hairpin RNA (hpRNA) of CAD gene, under the control of 35S CaMV promoter, was used to silence CAD expression (Wróbel-Kwiatkowska et al., 2007).

A severe reduction in secondary wall thickness of fibers, but not that of xylem vessels, as it occurs in the *Arabidopsis WALLS ARE THIN 1* (*wat1*) mutant, also increased resistance to vascular plant pathogens, such as the bacteria *R. solanacearum* and *Xanthomonas campestris* pv. *campestris*, the fungi *Verticillium dahliae* and *Verticillium alboatrum*, and the necrotrophic fungus *P. cucumerina* (Denancé et al., 2013). *WAT1* encodes a tonoplast localized indole acetic acid (auxin) transporter (Pesquet et al., 2005; Ranocha et al., 2010, 2013). Auxin content was found to be lower in roots, but not in leaves of the *wat1* mutant than in those of wild-type plants. In contrast, salicylic acid content was higher in the roots of the *wat1* mutant than in those of wild-type plants. Introduction in *wat1* plants of *NahG*, the bacterial gene coding for a salicylic acid-degrading hydroxylase, restored full susceptibility to the bacteria (Denancé et al., 2013). These data and those obtained by comparative transcriptomic analyses of *wat1* and wild-type plants suggest that *wat1*-mediated resistance is again not caused by altering the strength of the wall as a passive barrier, but that it is dependent on the activation of immune responses, mainly localized in the vascular system, which are partially dependent on the salicylic acid pathway. This defense response has been described as “vascular immunity” (Denancé et al., 2013).

Alteration of glucoronoxylans and xyloglucans or modifications in the content of wall xylose, which is the major sugar component of these polysaccharides, also impacts resistance to pathogens in *Arabidopsis*. For example, plants with enhanced levels of wall-bound xylose, as it occurs in the *de-etiolated3* (*det3*) and *irx6* mutants (Brown et al., 2005; Rogers et al., 2005) or with alterations in the structure of xyloglucan, as in the *xyl1-2* mutant (Sampedro et al., 2010), show an enhanced resistance to the necrotrophic fungus *P. cucumerina* (Delgado-Cerezo et al., 2012; **Table 1**). In contrast, impairment of the *ERECTA* (*ER*) gene encoding a PRR resulted in a reduced content of xylose besides other cell wall alterations in *Arabidopsis* (Sánchez-Rodríguez et al., 2009). The *er* mutant was found to be more susceptible than wild-type plants to several pathogens, such as the necrotrophic fungus *P. cucumerina*, the vascular bacterium *R. solanacearum* and the vascular oomycete *Pythium irregulare* (**Table 1**; Godiard et al., 2003; Llorente et al., 2005; Adie et al., 2007). The enhanced susceptibility to *P. cucumerina* and the cell wall features of the *er* mutant, including its reduced xylose content, were restored to wild-type levels by mutations in *SUPPRESSOR OF ERECTA 1* and 2 (*SER1* and *SER2)*, further suggesting a link between cell wall xylose content and resistance to pathogens (Sánchez-Rodríguez et al., 2009). Although several defense genes are constitutively up-regulated in the *ser1* and *ser2* mutants, the precise molecular basis of their resistance has not yet been fully elucidated and the *SER* genes have not been characterized yet (Sánchez-Rodríguez et al., 2009). *Arabidopsis* mutants in the Gβ and Gγ1/γ2 subunits of the heterotrimeric G protein (i.e., *agb1* single and *agg1 agg2* double mutants, respectively) also have a reduced content of xylose in their cell walls and are hypersusceptible to the necrotrophic fungi *P. cucumerina* and *Alternaria brassicicola*, the biotrophic bacterium *P. syringae* and the vascular fungus *Fusarium oxysporum* (**Table 1**; Llorente et al., 2005; Trusov et al., 2010; Klopﬄeisch et al., 2011; Delgado-Cerezo et al., 2012; Liu et al., 2013; Lorek et al., 2013; Torres et al., 2013). Interestingly, the reduced resistance of *agb1* single and *agg1 agg2* double mutants was found to be independent of defense pathways required for resistance to these pathogens, such as those regulated by abscisic acid, salicylic acid, jasmonic acid and ethylene, and those that regulate the biosynthesis of tryptophan-derived metabolites (Delgado-Cerezo et al., 2012; Lorek et al., 2013; Torres et al., 2013). It has been suggested that the reduced resistance in the *agb1* and *agg1 agg2* mutants is rather the direct consequence of a weakened cell-wall and a defective production of reactive oxygen species (ROS) upon pathogen infection (Delgado-Cerezo et al., 2012; Jiang et al., 2012; Liu et al., 2013; Lorek et al., 2013). Together, these data suggest that shifts in the xylose content of the cell wall, e.g., by altering the glucoronoxylan and xyloglucan content, are responsible, at least in part, for the altered susceptibility of some *Arabidopsis* secondary cell wall mutants to pathogens.

Cell wall polysaccharides such as xylan, (gluco)mannan and xyloglucan can be acetylated. Four *Reduced Wall Acetylation* genes (*RWA1–RWA4*) are involved in the acetylation of xylan during secondary wall biosynthesis. The expression of these genes is regulated by SND1, a transcriptional master switch of secondary wall biosynthesis (Lee et al., 2011). Remarkably, the *Arabidopsis rwa2* mutant, that has ∼20% lower levels of polysaccharide *O*-acetylation but no obvious alteration in growth and development, is more resistant than wild-type plants to the necrotrophic fungus *B. cinerea* (Manabe et al., 2011). The relevance of the degree of xylan acetylation in plant resistance to pathogens is further supported by the enhanced resistance to the necrotrophic fungi *B. cinera* and *Bipolaris sorkiniana* of transgenic *Arabidopsis* and *Brachypodium distachyon*, respectively, that have a reduced xylan acetylation due to overexpression of a xylan acetylesterase from *Aspergillus nidulans* (*AnAXE*; **Table 1**; Pogorelko et al., 2013). These data indicate that the degree of acetylation of specific secondary cell wall polymers might be a determinant of susceptibility to particular pathogens. In addition to RWA proteins, members of the trichome birefringence (TBR) and TBR-like (TBL) protein families are also involved in the *O*-acetylation of wall polysaccharides (Gille et al., 2011). The *Arabidopsis powdery mildew resistant5* (*pmr5*) mutant, impaired in a TBL member, has a decrease in cell-wall esterification as demonstrated by Fourier transform infrared (FTIR) analysis, but it has yet to be demonstrated whether *pmr5* cell walls have an altered polysaccharide *O*-acetylation (Vogel et al., 2004; Gille and Pauly, 2012). The *pmr5* mutant is more resistant than wild-type plants to powdery mildew fungi (i.e., *E. cichoracearum* and *E. orontii),* whereas its resistance to the bacterium *P. syringae* or the oomycete *Peronospora parasitica* (re-named *Hyaloperonospora arabidopsidis)* was similar to that of wild-type plants (Vogel et al., 2004). Taken together, these data indicate that a decrease in cell wall acetylation in *Arabidopsis* resulted in an enhanced resistance to several fungi, but the molecular mechanisms explaining this resistance phenotype have yet to be elucidated.

Primary cell wall remodeling can also impact pathogen resistance, as exemplified by the enhanced resistance to some pathogens of mutants defective in the CESA subunits required for primary cell wall cellulose biosynthesis (i.e., *ixr1/cev1*; Ellis et al., 2002; Hernández-Blanco et al., 2007). Similarly, modification of the biosynthesis and/or structure (e.g., degree of methylesterification or acetylation) of wall pectins can affect pathogen resistance (Vogel et al., 2002, 2004; Lionetti et al., 2007; Raiola et al., 2011; Volpi et al., 2011; Bethke et al., 2014). The complex contribution of pectin amount/structure to the regulation of plant innate immunity has been nicely reviewed in several recent publications that also describe the different virulence mechanism used by pathogens to modify or degrade pectins in order to favor plant colonization (Ferrari et al., 2012; Lionetti et al., 2012; Bellincampi et al., 2014).

## PHENYLPROPANOID AND LIGNIN BIOSYNTHESIS

Lignin is an aromatic polymer that is mainly deposited in secondary thickened cell walls where it provides strength and imperviousness. In monocot and dicot plants, lignin is mainly made from the monolignols coniferyl and sinapyl alcohol that give rise to the guaiacyl (G) and syringyl (S) units in the lignin polymer, respectively. *p*-Coumaryl alcohol, that gives rise to the *p*-hydroxyphenyl (H) units in the lignin polymer, is a minor monolignol that is slightly more abundant in monocot than in dicot cell walls. Lignin of gymnosperms is typically composed of G units and low levels of H units, but lacks S units. In several plant species, the traditional monomers are incorporated into the lignin in acylated forms. For instance, kenaf lignin is rich in sinapyl acetate-derived units, lignin of grasses has a high content of sinapyl *p*-coumarate-derived lignin units and poplar lignin incorporates sinapyl *p*-hydroxybenzoate (Morreel et al., 2004; Del Río et al., 2007; Lu and Ralph, 2008; Hatfield et al., 2009). In addition, plants do accept a range of other phenolics as lignin monomers. For example, lignin in wheat straw has relatively high levels of the flavonoid tricin (Del Río et al., 2012), whereas phenylpropanoid aldehydes and acids are found in the lignin of a range of wild-type and genetically engineered plants (Kim et al., 2000; Dauwe et al., 2007; Vanholme et al., 2012a; Van Acker et al., 2013, 2014).

**FIGURE 1:**
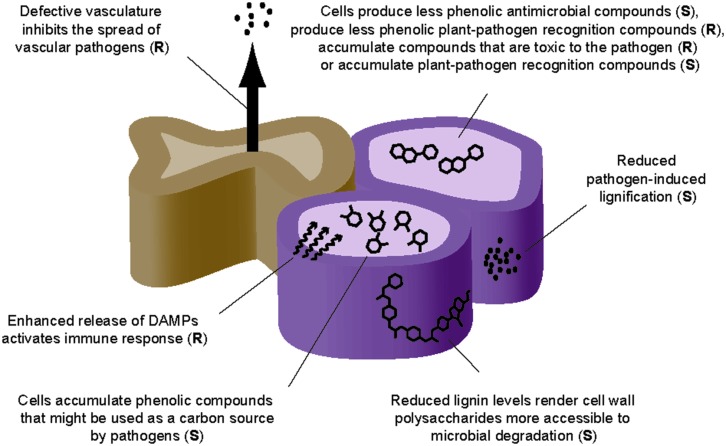
**The phenylpropanoid and monolignol biosynthetic pathway in *Arabidopsis* (adapted from Vanholme et al., 2010, 2013)**.

The lignin biosynthetic pathway is relatively well described in many species including *Arabidopsis*, tobacco, alfalfa and poplar, and is generally divided in two branches: (i) the general phenylpropanoid pathway from Phe to feruloyl-CoA and (ii) the monolignol-specific pathway from feruloyl-CoA to the monolignols (**Figure 1**). At least eleven enzymes are involved in the biosynthesis of the monolignols from Phe: phenylalanine ammonia-lyase (PAL), cinnamate 4-hydroxylase (C4H), 4-coumarate:CoA ligase (4CL), hydroxycinnamoyl-CoA shikimate/quinate hydroxycinnamoyl transferase (HCT), *p*-coumarate 3-hydroxylase (C3H), caffeoyl shikimate esterase (CSE), caffeoyl-CoA *O*-methyltransferase (CCoAOMT), cinnamoyl-CoA reductase (CCR), ferulate 5-hydroxylase (F5H), caffeic acid *O*-methyltransferase (COMT), and cinnamyl alcohol dehydrogenase (CAD; **Figure 1**; Boerjan et al., 2003; Bonawitz and Chapple, 2010; Vanholme et al., 2010, 2013). After their biosynthesis, the monolignols are transported to the cell wall where they are oxidized by laccases and/or peroxidases to monolignol radicals. Subsequently, these monolignol radicals couple in a combinatorial fashion with the formation of various types of chemical bonds of which the ether (8-O-4), resinol (8–8), and coumaran (8–5) bonds are the most prominent ones (Boerjan et al., 2003; Ralph et al., 2004; Vanholme et al., 2012a).

The biosynthesis and deposition of lignin in secondary cell walls is developmentally programmed and it is generally accepted that lignin provides a physical barrier against initial pathogen colonization (Buendgen et al., 1990; Bonello et al., 2003). In addition, lignin or lignin-like phenolic polymers are synthesized and rapidly deposited in cell walls in response to biotic and abiotic stresses and to cell wall structure perturbations (Caño-Delgado et al., 2003; Tronchet et al., 2010; Sattler and Funnell-Harris, 2013). The deposition of lignin in infected cells may prevent the spread of toxins and enzymes of the pathogen into the host and at the same time also the transfer of water and nutrients from the host cells to the pathogen (Smith et al., 2007). Both biotic and abiotic stresses have been shown to induce the expression of genes of the phenylpropanoid pathway in different tissues and cell cultures of several plants species, resulting in the enhanced accumulation of the corresponding enzymes, increased enzymatic activities and wall lignification (Kliebenstein et al., 2002; Bhuiyan et al., 2007; Zhao et al., 2009). For example, in Chinese cabbage (*Brassica rapa L.* ssp. *pekinensis)* infected with the necrotrophic bacterium *Erwinia carotovora* subsp. *carotovora*, transcriptomic analyses revealed that 12 genes putatively encoding enzymes involved in lignin biosynthesis were up-regulated (Zhang et al., 2007), and in *Camelina sativa* the expression of *CsCCR2* was induced after inoculation with the necrotrophic fungus *Sclerotinia sclerotiorum* (Eynck et al., 2012). In cell suspension cultures of *Linum usitatissimum* treated with different fungal PAMPs, the expression of genes encoding PAL, CCR, and CAD was elevated, PAL activity was enhanced and monolignol-derived compounds accumulated (Hano et al., 2006). Similarly, in suspension cultured cells of a blast-resistant rice genotype (*Oryza sativa* L. cv. Gigante Vercelli) treated with cell wall hydrolysates from the fungal pathogen *Magnaporthe oryzae*, up-regulation of *PAL* genes was observed (Giberti et al., 2012). Also in wheat lignification acts as a defense response against pathogens: for example, S-rich lignin was found to accumulate during the hypersensitive reaction of wheat to *Puccinia graminis* infection (Menden et al., 2007), and S lignin was made in wheat sheath epidermal cells infected with *F. proliferatum* (Bishop et al., 2002). In contrast, no changes in the lignin content occurred in wheat leaves infected with Wheat Streak Mosaic Virus (Kofalvi and Nassuth, 1995).

In addition to its function in the biosynthesis of lignin, the phenylpropanoid pathway is required for the synthesis of numerous other phenolic compounds, such as stilbenes, coumarins, (neo-)lignans, phenylpropanoid conjugates, and flavonoids (Lo and Nicholson, 1998; Yu et al., 2000, 2005; Dixon et al., 2002; Naoumkina et al., 2010). Many of these compounds are considered to be phytoalexins, i.e., antimicrobial compounds implicated in plant defense (Daayf et al., 2012; König et al., 2014). Therefore, impairing steps of the phenylpropanoid pathway can result in either accumulation or reduced abundance of these compounds, often resulting in pleiotropic effects on plant resistance (Weiergang et al., 1996; Ruuhola and Julkunen-Tiitto, 2003; Dicko et al., 2005; Pan et al., 2006; Lozovaya et al., 2007). Phenolic compounds are also important in plant-pathogen recognition. For example, the expression of *Agrobacterium tumefaciens Virulence* (*Vir*) genes, needed for infection, is induced by phenolic compounds and infection of the host plant cannot take place in the absence of these compounds (Lee et al., 1992, 1995; Maury et al., 2010).

## EFFECT OF PHENOLIC CONTENT AND LIGNIN MODIFICATIONS ON PATHOGEN RESISTANCE

Evidence for a role for lignin and soluble phenolics in plant defense has been obtained from the analysis of the pathogen resistance of transgenic plants and mutants with contrasting lignin amount or composition. For example, in tomato, the total content of soluble phenolics and lignin were significantly higher in varieties that were resistant to the vascular bacterium *R. solanacearum* than in susceptible ones, and this enhanced resistance was associated to a greater accumulation of lignin in roots upon bacterial infection, a process that was triggered by salicylic acid (Mandal et al., 2011, 2013). In tobacco, plants down-regulated for *PAL* had reduced levels of chlorogenic acid and exhibited more rapid and extensive lesion development than wild-type plants upon infection with the fungal pathogen *Cercospora nicotianae* (Maher et al., 1994). The increased disease susceptibility in this *PAL*-suppressed line was suggested not to result from the inhibition of the pathogen-induced response, but rather from the decrease in the developmental accumulation of chlorogenic acid (Maher et al., 1994). However, the lignin content in this *PAL*-suppressed line was not determined and therefore it cannot be excluded that the enhanced susceptibility was caused by reduced lignin content or a weaker cell wall (Maher et al., 1994). In accordance with the latter studies, transgenic tobacco plants constitutively overexpressing *PAL* genes showed a higher tolerance toward *C. nicotianae* and *Phytophthora parasitica* pv. *nicotianae* (Way et al., 2002, 2011; Shadle et al., 2003). Notably, *COMT* and *CCoAOMT* antisense tobacco lines were more resistant to *Agrobacterium tumefaciens* infection and showed a reduced tumor area and mass relative to wild-type plants (Maury et al., 2010). The phenolic compounds secreted by these antisense plants upon wounding did not induce the expression of the bacterial *Vir* genes equally well as those secreted from wounded wild-type plants (Maury et al., 2010). In other words, the *Agrobacterium* did not recognize its host because of the difference in soluble phenolics.

In cotton (*Gossypium hirsutum*), quantitative analysis of resistance to the wilt fungus *V. dahliae* revealed an association between increased lignification in the stems upon infection and resistance against wilt (Xu et al., 2011). In line with these data, overexpression of the cotton *DIRIGENT1* gene, which enhances lignification, blocks the spread of *V. dahliae* (Shi et al., 2012). In alfalfa (*Medicago sativa*), down-regulation of the *HCT* gene leads to plants with reduced lignin levels, constitutive defense responses and enhanced tolerance to the fungal pathogen *Colletotrichum trifolii*. This activation of defense responses was hypothesized to be triggered by bioactive cell wall fragments released from the secondary cell wall (Gallego-Giraldo et al., 2011b). In melon (*Cucumis melo*), lignin accumulation upon infection was found to increase faster and to a higher level in lines resistant to the powdery mildew fungus *Podosphaera fusca* than in susceptible lines, and this differential accumulation correlated with enhanced PAL levels (Romero et al., 2008). Lignin composition seems to play an important role in pathogen resistance in flax, as RNAi-mediated suppression of a *CAD* gene increased flax susceptibility to the vascular fungus *F. oxysporum* (Wróbel-Kwiatkowska et al., 2007).

Also in grasses the effect of modifying lignin biosynthesis on plant susceptibility has been investigated. For instance, in wheat *(Triticum monococcum)*, silencing the monolignol biosynthesis genes *TmPAL*, *TmCOMT*, *TmCCoAOMT*, and *TmCAD* led to super-susceptibility of leaf tissues to the fungus *B. graminis* f. sp. *tritici*, the causal agent of powdery mildew disease (Bhuiyan et al., 2009). The increased accumulation of mono- and diferulates in the cell walls of oat and wheat upon infection with *P. coronate* sp. *avenae* and *Agrobacterium* sp., respectively, has been associated with resistance toward these pathogens (Ikegawa et al., 1996; Parrott et al., 2002). In transgenic rice overexpressing the *NPR1 HOMOLOG 1* (*NH1*), a suppressor mutant screening was performed and a mutation in the *SUPPRESSSOR OF NH1-MEDIATED LESION FORMATION AND RESISTANCE* (*SNL6*) gene, which encodes a CCR-like protein, was selected. *snl6* mutants had a lower lignin content and a reduced resistance to the bacterium *X. oryzae* pv. *oryzae* (Bart et al., 2010). Mutations in *BROWN MIDRIB 6* (*BMR6*) and *BMR12* in sorghum (*Sorghum bicolor* L.) allowed the development of forage and grain lines with a reduced lignin content and modified lignin composition (Oliver et al., 2005). The *bmr6* and *bmr12* mutants, that are defective in CAD and COMT proteins, respectively, restricted the growth of different *Fusarium* spp. (*F. thapsinum*, *F. proliferatum*, and *F. verticillioides*), but not that of *Gibberella fujikuroi* (Bout and Vermerris, 2003; Sattler et al., 2009; Funnell-Harris et al., 2010). It is unknown whether the alteration of lignin composition or the accumulation of phenolic compounds is causative to the enhanced resistance of these sorghum mutants to *Fusarium* sp.

In trees, the contribution of lignin amount or composition on susceptibility to pathogens has been also investigated. For example, in eucalyptus, the deposition of lignin in necrophylatic periderm in the early stages of infection by *Mycosphaerella* explains the greater resistance of *Eucalyptus nitens* as compared with *E. globulus* (Smith et al., 2007). Comparative metabolite profiling of xylem tissue of *Ulmus minor* and *Ulmus minor × Ulmus pumila* after inoculation with *Ophiostoma novo-ulmi* showed that the hybrid has a faster defense response, which is characterized by an increase in the amount of lignin (Martin et al., 2007). Similarly, the infection of *Pinus nigra* by *Sphaeropsis sapinea* induces an increase in the deposition of lignin that was associated to resistance (Bonello and Blodgett, 2003). Interestingly, in hybrid poplar (*Populus tremula* ×* Populus alba*) no increased disease incidence was observed in field-grown antisense *COMT* and *CAD* lines relative to that observed in wild-type trees, nicely showing that altered lignin biosynthesis does not necessarily negatively impact resistance to pathogens (Pilate et al., 2002; Halpin et al., 2007).

In summary, a general positive correlation between lignin amount and pathogen resistance has been observed, in particular when the plant-pathogen interaction concerns vascular pathogens, such as *Fusarium* sp., *Xanthomonas* sp. or *Verticilium* sp., that generally spread through the secondary-thickened xylem. In the majority of the examples analyzed, the impact of lignin modification on the regulation of other defense responses has not been studied, and it is not yet possible to conclude whether the role of lignin in resistance is merely passive or active by regulating specific immune responses.

## CONTRIBUTION OF LIGNIN TO PATHOGEN RESISTANCE IN *Arabidopsis*

Several lines of evidence support a role for lignin in immunity of *Arabidopsis* to pathogens. The expression of some lignin-biosynthesis genes was induced, and the amount of lignin increased, by treating *Arabidopsis* with hormones (i.e., salicylic acid, abscisic acid or jasmonic acid) that regulate plant defense (Mohr and Cahill, 2007; Chen et al., 2009; Gallego-Giraldo et al., 2011a). Similarly, infection of *Arabidopsis* with particular pathogens, such as the bacteria *P. syringae* pv. *tomato* and *X. campestris,* resulted in increased expression of lignin-biosynthesis genes and in higher lignin levels (Mohr and Cahill, 2007; Quentin et al., 2009). Experiments also hinted to specific stress-related roles for the different gene family members involved in certain enzymatic conversions. For example, the *Arabidopsis CCR2* gene has been suggested to participate in the hypersensitive response to *X. campestris* as its expression was up-regulated after inoculation with this bacterium, in contrast to *CCR1* which was preferentially expressed during development (Lauvergeat et al., 2001).

The analysis of *Arabidopsis* mutants defective in lignin biosynthesis and of transgenic plants overexpressing lignin biosynthesis genes has contributed to unravel the role of lignin in plant immunity. For example, two *pal1/2/3/4* quadruple mutants with 20% and 25% residual lignin levels and 25% residual salicylic acid levels, showed a stunted growth and were hypersusceptible to *P. syringae*. In addition, the total salicylic acid levels in the quadruple mutants after infection were about 50% of those in wild type, suggesting that pathogen-induced, salicylic acid-mediated resistance might be partially impaired in this mutant (Huang et al., 2010). The *Arabidopsis comt* mutant was found to be slightly more susceptible than wild-type plants to *P. syringae* pv. *tomato DC3000*, but also to *B. cinerea*, *A. brassicicola* and *X. campestris* pv. *campestris*, and *B. graminis* f. sp. *hordei*, that is a barley pathogen that does not colonize *Arabidopsis* plants (Quentin et al., 2009). Unexpectedly, asexual sporulation of the oomycete *H. arabidopsidis*, causing the downy mildew disease, was impaired in the *comt* mutant (Quentin et al., 2009). This phenotype was not correlated with an increased salicylic and jasmonic acid-dependent defense, but with a higher frequency of oomycete sexual reproduction within *comt* mutant tissues (Quentin et al., 2009). It was further proven that *comt* mutants accumulated soluble 5-hydroxyferuloyl-malate and that this compound promoted sexual oomycete reproduction *in vitro* (Quentin et al., 2009). *Arabidopsis f5h1* mutants showed an increased susceptibility to the fungal pathogen *S. sclerotiorum* and to the vascular fungus *Verticillium longisporum* (Huang et al., 2009; König et al., 2014). The *f5h1* mutants have similar amounts of lignin as compared to wild type, but lack S units in the lignin and are sinapate ester deficient (Meyer et al., 1998; Vanholme et al., 2012b; König et al., 2014). Because sinapate esters inhibit fungal growth *in vitro*, their absence in *f5h1* might explain the mutant’s increased susceptibility toward fungal pathogens (König et al., 2014). Interestingly, the *UGT72E2* over-expressing *Arabidopsis* line, in which lignin was not altered but the soluble phenylpropanoid coniferin accumulated, was less susceptible to *V. longisporum* (König et al., 2014). All together these results again strongly support that not only the lignin polymer but also the soluble phenolic pool plays a significant role in the defense of plants against pathogens. As described above for crops and other plant species, a general positive correlation between lignin amount and resistance toward pathogens has been observed in *Arabidopsis* too. However, a deeper characterization of the impact of the lignin alterations on *Arabidopsis* immune responses and its relation with the content and profile of phenolics is needed to elucidate the molecular mechanisms explaining the differential responses of the mutants to pathogen infection (**Table 1**).

In *Arabidopsis*, the plant’s response to the perturbation of lignin has been studied in a collection of mutants, each mutated in a single gene of this pathway, by combining transcriptomics and metabolomics (Vanholme et al., 2012b; **Figure 1**). These analyses revealed that *c4h*, *4cl1*, *ccoaomt1*, and *ccr1* mutants, that produced less lignin, upregulated the shikimate, methyl-donor, and phenylpropanoid pathways (i.e., the pathways supplying the monolignols), whereas, *f5h1* and *comt* mutants, that provoked lignin compositional shifts, downregulated the very same pathways (Vanholme et al., 2012b). Moreover, some of these mutant alleles revealed subtle differences in the metabolic and gene expression profiles that might contribute to differential resistance responses to pathogens (Vanholme et al., 2012b). This collection of mutants represents a unique tool to further characterize the specific contribution of lignin biosynthesis to resistance against different types of pathogens in *Arabidopsis*.

## EFFECTS OF MODIFYING LIGNIN AND SECONDARY CELL WALL STRUCTURE ON PLANT PATHOGEN RESISTANCE: HYPOTHESES AND OPEN QUESTIONS

**FIGURE 2:**
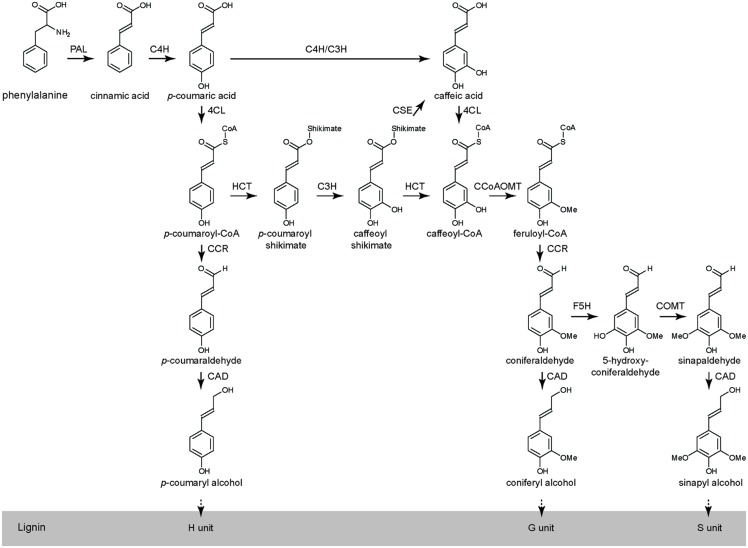
**Model illustrating lignin and secondary cell wall modifications resulting in enhanced resistance (R) or enhanced susceptibility (S) to pathogens**.

Several hypotheses can be formulated to explain the resistance or the susceptibility observed in mutants and transgenic plants affected in the amount and/or composition of lignin and, more broadly, the secondary cell wall (**Figure 2**): (i) The perturbation of lignin or secondary cell wall structure modifies the physical barrier that pathogens must overcome to invade the plant. This perturbation can lead to enhanced resistance or susceptibility as these pathogens might lack the enzymes required for a proper degradation of this novel physical barrier, or wall degradation by pathogens is now facilitated, respectively; (ii) The reduced amount of lignin or the modification of lignin or secondary cell wall composition impacts the strength of the secondary cell wall resulting in collapsed xylem. A drop in vascular conduction might negatively contribute to plant colonization by vascular pathogens; (iii) The reduction of lignin amount and the modification of the secondary cell wall can loosen the wall, facilitating the constitutive or pathogen-induced release of cell wall DAMPs, which might trigger immune responses resulting in enhanced resistance to pathogens; (iv) The perturbation of the lignin pathway could lead to the accumulation of soluble phenolic compounds that are either toxic to some pathogens (e.g., resulting in a reduced virulence), or serve as a new carbon or nutrient source for pathogens that then will grow better (e.g., resulting in enhanced virulence); (v) Similarly, the perturbation of the lignin pathway could also lead to the accumulation or decrease of soluble phenolics that are plant-pathogen recognition compounds, which would result in an enhanced susceptibility or resistance, respectively. Although these hypotheses might explain some of the published phenotypes, other molecular explanations cannot be excluded and a deeper molecular and biochemical characterization is required for a better understanding of the contribution of the secondary cell wall to pathogen resistance.

## PERSPECTIVES

Lignin negatively impacts the conversion of lignocellulosic biomass into fermentable sugars, making it one of the most important limiting factors in the processing of plant biomass to pulp and biofuels (Chen and Dixon, 2007; Dien et al., 2009; Van Acker et al., 2013). Hence, modifications of the plant secondary cell wall can contribute to the improvement of biomass processing in paper mills and bio-refineries. However, one critical question for lignocellulosic feedstock development is whether engineering the secondary cell wall, including its lignin content and composition, will affect plant defense against pathogens. In this review, we have summarized the role of the cell wall in plant resistance toward pathogens. We conclude that plants with altered secondary cell walls may either have an enhanced or a reduced resistance toward pathogens, or no effect at all, depending on the alterations made and the pathogens tested. Because our current knowledge on the role of the cell wall (primary or secondary) in defense against pathogens is still fragmentary, it is difficult to predict how specific alterations of the cell wall will influence a plant’s resistance toward pathogens. A deeper investigation of the role of the plant cell wall in pathogen resistance and the biochemical networks underlying this resistance is required.

## Conflict of Interest Statement

The authors declare that the research was conducted in the absence of any commercial or financial relationships that could be construed as a potential conflict of interest.
